# Community Factors Associated With Telemedicine Use During the COVID-19 Pandemic

**DOI:** 10.1001/jamanetworkopen.2021.10330

**Published:** 2021-05-18

**Authors:** Sadiq Y. Patel, Sherri Rose, Michael L. Barnett, Haiden A. Huskamp, Lori Uscher-Pines, Ateev Mehrotra

**Affiliations:** 1 Department of Health Care Policy, Harvard Medical School, Boston, Massachusetts; 2 Center for Health Policy, Stanford University, Stanford, California; 3 Division of General Internal Medicine and Primary Care, Department of Medicine, Brigham and Women’s Hospital, Boston, Massachusetts; 4 Department of Health Policy and Management, Harvard T. H. Chan School of Public Health, Boston, Massachusetts; 5 RAND Corporation, Arlington, Virginia; 6 Division of General Medicine, Beth Israel Deaconess Medical Center, Boston, Massachusetts; 7 OptumLabs Visiting Fellow, Eden Prairie, Minnesota

## Abstract

This cross-sectional study investigates which community factors may be associated with the increase in telemedicine use during the COVID-19 pandemic.

## Introduction

While telemedicine use grew rapidly during the COVID-19 pandemic, there was substantial geographic variation in uptake.^1,2^ What drives this variation is unclear. To understand drivers of telemedicine use, we examined the association of county-level telemedicine use with community factors among individuals with commercial or Medicare Advantage insurance.

## Methods

Using OptumLabs Data Warehouse deidentified medical claims, we captured all outpatient visits, in person and telemedicine, from January 1 to July 14, 2020. We defined outpatient visits using Medicare’s list of *Current Procedural Terminology (CPT) *codes eligible for telemedicine.^3^ Telemedicine visits were those with modifier codes GT, GQ, or 95, or *CPT* codes 99441 to 99443 with the remainder defined as in-person visits.

After excluding counties with fewer than 100 members enrolled from July 2019 through July 2020, we assessed county-level telemedicine use during the pandemic (defined as March 18 to July 14, 2020) using the percentage of total visits delivered via telemedicine. We measured county-level population density (number of people per square mile) as a proxy for rurality, race, income, broadband availability, and per capita number of hospital beds, community health centers, physicians, and advanced practice registered nurses using data from the US Census and Area Health Resource Files.^4^ County-level prepandemic telemedicine use was measured as the percentage of total visits delivered via telemedicine from January 1 to March 17, 2020, and cumulative COVID-19 cases per capita were measured over 2 periods, March 18 to April 16, 2020, and March 18 to July 14, 2020.

To measure the association between these county characteristics and percentage point differences in telemedicine use, we used targeted maximum likelihood estimation, a machine learning procedure used in prior clinical literature.^5^ We divided county characteristic measures into tertiles for meaningful interpretation except for those where there were positivity violations. We addressed violations by dichotomizing variables (see eAppendix in the Supplement). The Harvard Medical School institutional review board exempted this study from review because it involved the study of data recorded such that participants cannot be reasonably identified. This study adheres to the Strengthening the Reporting of Observational Studies in Epidemiology (STROBE) reporting guidelines for cross-sectional studies.

Data analyses were performed using R statistical software version 3.6.3 (R Project for Statistical Computing) with the SuperLearner and tmle packages.^5^ These analyses were performed on September 17, 2020.

## Results

Across 2800 counties with at least 100 enrollees, the mean (SD) and median (interquartile range [IQR]) percentage of total visits delivered via telemedicine were 19.6% (8.3%) and 18.6% (13.3%-24.7%), respectively. The median (IQR) percentage of telemedicine visits in the 5 quintiles of counties was 10.0% (8.0%-11.2%) for quintile 1, 14.3% (13.3%-15.4%) for quintile 2, 18.6% (17.6%-19.8%) for quintile 3, 23.2% (22.1%-24.9%) for quintile 4, and 31.0% (28.5%-35.2%) for quintile 5. (Figure).

**Figure.**
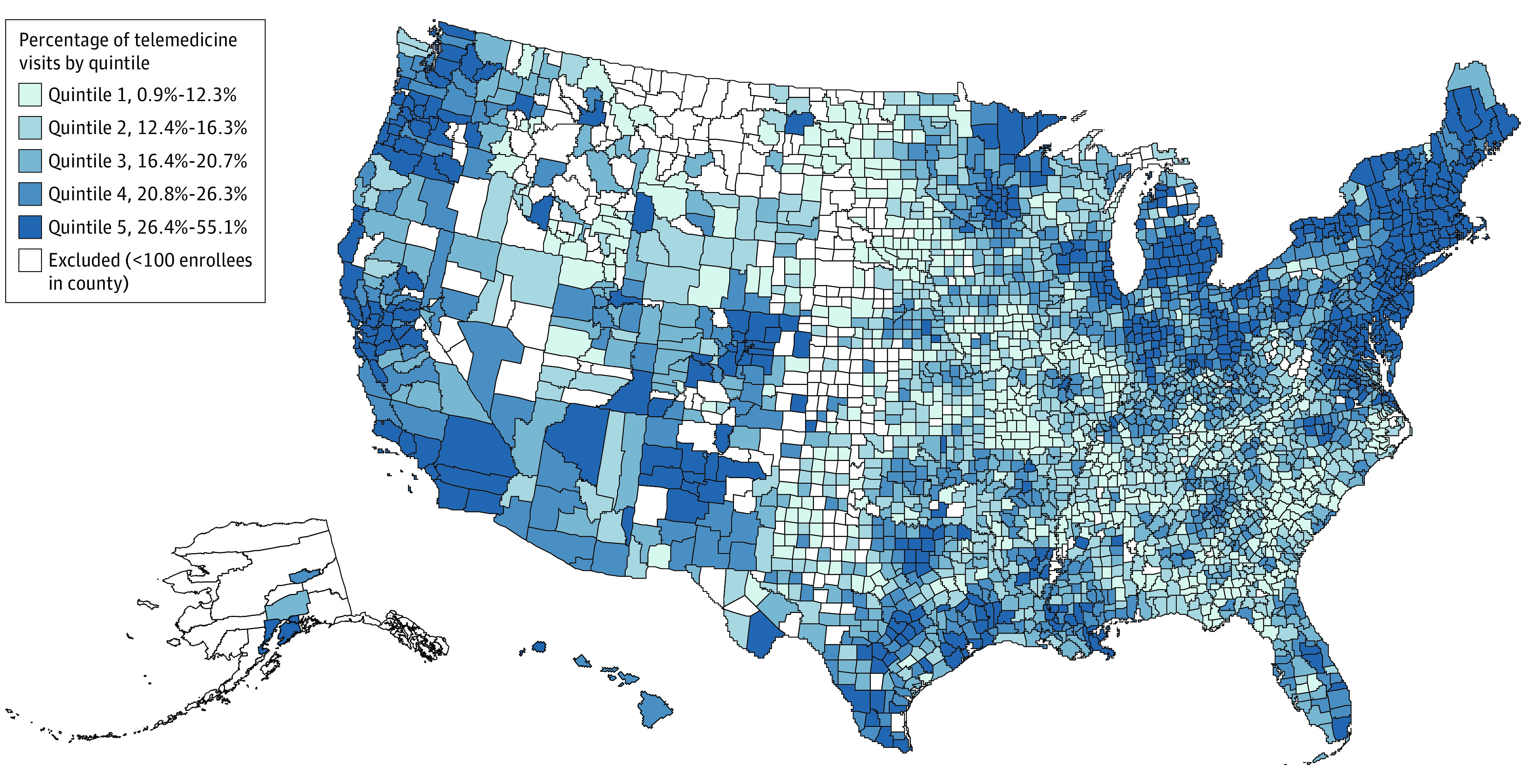
US Geographic Variation in the County-Level Percentage of Totals Visits Delivered by Telemedicine During COVID-19 Percentage of total visits delivered by telemedicine is based on visits during the COVID-19 pandemic (defined here as March 18 to July 14, 2020). Quintiles based on the distributions across the 2800 US counties in our sample.

We observed less telemedicine use in counties with lower median income (difference of below vs above median: 2.5%; 95% CI, 2.2%-2.7%), lower population density (difference of lowest vs highest tertile: 7.5%; 95% CI, 7.2%-7.8%), less broadband availability (difference of below vs above median: 1.6%; 95% CI, 1.3%-1.8%), less prepandemic telemedicine use (difference of highest vs lowest tertile: 2.6%; 95% CI, 2.2%-2.9%), and fewer COVID-19 cases per capita in the first 30 days of pandemic (difference of highest vs lowest tertile: 2.8%, 95% CI, 2.5%-3.2%) (Table). We did not observe an association between telemedicine use and COVID-19 incidence over a longer period through July 14.

**Table.** Targeted Maximum Likelihood Estimation Analysis of the Association Between the Percentage of Total Visits Delivered via Telemedicine During COVID-19 and Community Factors^a^

Characteristic	Counties, No.	Percentage point change in proportion of total telemedicine visits (95% CI)	
		First 30 d^b^	First 17 wk^c^
Total visits per 1000 members in pre-COVID period, tertile			
Low (<1205 visits per capita)	932	[Reference]	[Reference]
Middle	934	−0.09 (−0.49 to 0.32)	−0.12 (−0.53 to 0.28)
High (>1508 visits per capita)	934	−1.45 (−1.77 to −1.13)	−1.20 (−1.57 to −0.84)
% of total visits via telemedicine in pre-COVID period, tertile			
Low (<0.3%)	933	[Reference]	[Reference]
Middle	934	2.14 (1.84 to 2.44)	2.41 (2.11 to 2.72)
High (>0.7%)	933	2.59 (2.24 to 2.94)	2.47 (2.09 to 2.85)
Median household income, binary			
Below median (<$50 224)	1400	[Reference]	[Reference]
Above median (≥$50 224)	1400	2.46 (2.20 to 2.72)	2.77 (2.47 to 3.07)
Population density (No. of people per square mile), tertile			
Low (<32 individuals)	932	[Reference]	[Reference]
Middle	934	1.95 (1.61 to 2.31)	2.41 (2.11 to 2.72)
High (>93 individuals)	934	7.46 (7.15 to 7.78)	7.87 (7.53 to 8.21)
% of households with White, non-Hispanic, tertile			
Low (<72%)	932	[Reference]	[Reference]
Middle	934	0.71 (0.30 to 1.13)	0.41 (0.07 to 0.75)
High (>90%)	934	0.89 (0.62 to 1.16)	−0.05 (−0.28 to 0.19)
% of households with broadband, binary			
Below median (<74%)	1399	[Reference]	[Reference]
Above median (≥74%)	1401	1.57 (1.30 to 1.84)	1.31 (1.03 to 1.59)
Cumulative COVID-19 cases per capita during first 30 d, tertile^b^			
Low (<0.2 cases per capita)	933	[Reference]	[Reference]
Middle	933	0.78 (0.41 to 1.16)	NA
High (>0.6 cases per capita)	934	2.82 (2.47 to 3.16)	NA
Cumulative COVID-19 cases per capita during first 17 wk, tertile^c^			
Low (<3)	933	[Reference]	[Reference]
Middle	933	NA	−0.20 (−0.60 to 0.20)
High (>7)	934	NA	−1.02 (−1.33 to 0.71)
No. of hospital beds per capita, binary			
Below median (<1.9)	1399	[Reference]	[Reference]
Above median (≥1.9)	1401	−0.36 (−0.64 to −0.07)	−0.27 (−0.58 to 0.04)
No. of community health centers per capita, tertile			
Low (<0.01)	933	[Reference]	[Reference]
Middle	934	1.94 (1.55 to 2.34)	2.22 (1.82 to 2.61)
High (>0.1 CHCs per capita)	933	1.60 (1.24 to 1.97)	1.76 (1.39 to 2.13)
No. of total MDs and APRNs per capita, tertile			
Low (<1.1)	933	[Reference]	[Reference]
Middle	933	−0.24 (−0.56 to 0.09)	−0.23 (−0.60 to 0.14)
High (>2.1)	934	1.61 (1.30 to 1.91)	1.43 (1.10 to 1.76)

Abbreviations: APRN, advanced practice registered nurses; MD, medical doctor; NA, not applicable.

^a^ Targeted maximum likelihood estimation is an ensemble machine learning procedure used here for measures of association.

^b^ Cumulative COVID-19 cases during the first 30 days of the pandemic (through April 16, 2020).

^c^ Cumulative COVID-19 cases during the first 17 weeks of the pandemic (through July 14, 2020).

## Discussion

Through July 14, 2020, we observed substantial variation across counties in telemedicine use. Our results support concerns that rural and lower-income communities may be left behind in the shift to telemedicine use. To ensure telemedicine is accessible by all people in the US, interventions such as increased broadband investment in rural areas^6^ or greater reimbursement in disadvantaged communities may be needed.

Another key factor associated with less telemedicine use during the pandemic was prepandemic telemedicine use. Health care practitioners with the necessary technology and familiarity with technology may have been able to quickly increase telemedicine use.

Telemedicine adoption was also associated with incidence of COVID-19 over the first 4 weeks of the pandemic, but not incidence over a longer 16-week period. Possibly initial uncertainty about the virus in higher incidence areas drove uptake, and the urgency to use telehealth waned somewhat over time as communities became acclimated to the pandemic. A key limitation is that results may not generalize to other commercially insured populations and those with Medicare or Medicaid insurance.

## Notes

Supplement. **eAppendix.** Data Sources and Study Sample eReferences
