# Supplementary material for: Community Factors Associated With Telemedicine Use During the COVID-19 Pandemic
Source: JAMA Netw Open. 2021 May 18;4(5):e2110330. doi: 10.1001/jamanetworkopen.2021.10330 (PMC8132134; doi:10.1001/jamanetworkopen.2021.10330)
Supplement: Supplement. — eAppendix. Data Sources and Study Sample eReferences [file jamanetwopen-e2110330-s001.pdf]

Supplemental Online Content

Patel SY, Rose S, Barnett ML, Huskamp HA, Uscher-Pines L, Mehrotra A. Community factors associated with telemedicine use during the COVID-19 pandemic. *JAMA Netw Open.* 2021;4(5):e2110330. doi:10.1001/jamanetworkopen.2021.10330

eAppendix. Data Sources and Study Sample

eReferences

© 2021 Patel SY et al. *JAMA Network Open.*

eAppendix. Data Sources and Study Sample

Our study sample included de-identified claims for all outpatient visits from January 1, 2020 to July 14, 2020 in the OptumLabs Data Warehouse (OLDW), which includes de-identified medical claims and enrollment records for commercial enrollees offering a range of health plan products and currently covering roughly 13% of commercially insured US individuals nationally. We chose March 18th to define the start of COVID-19 because Medicare announced expanded telehealth coverage on March 17, 2020.[1] We included all plan enrollees with 13 months of continuous medical enrollment in any plan from July 2019 through July 2020. We excluded counties with fewer than 100 enrollees to obtain more stable measures of telemedicine use during the pandemic. Our concern was that in counties with a low number of enrollees, we would not be able to characterize telemedicine adoption. There could be a limited number of visits and a county could be labeled as "high" or "low" telemedicine uptake simply due to low sample of visits.

To help readers understand where the study population was located, the map below shows what fraction of the entire study population is located in a given county.

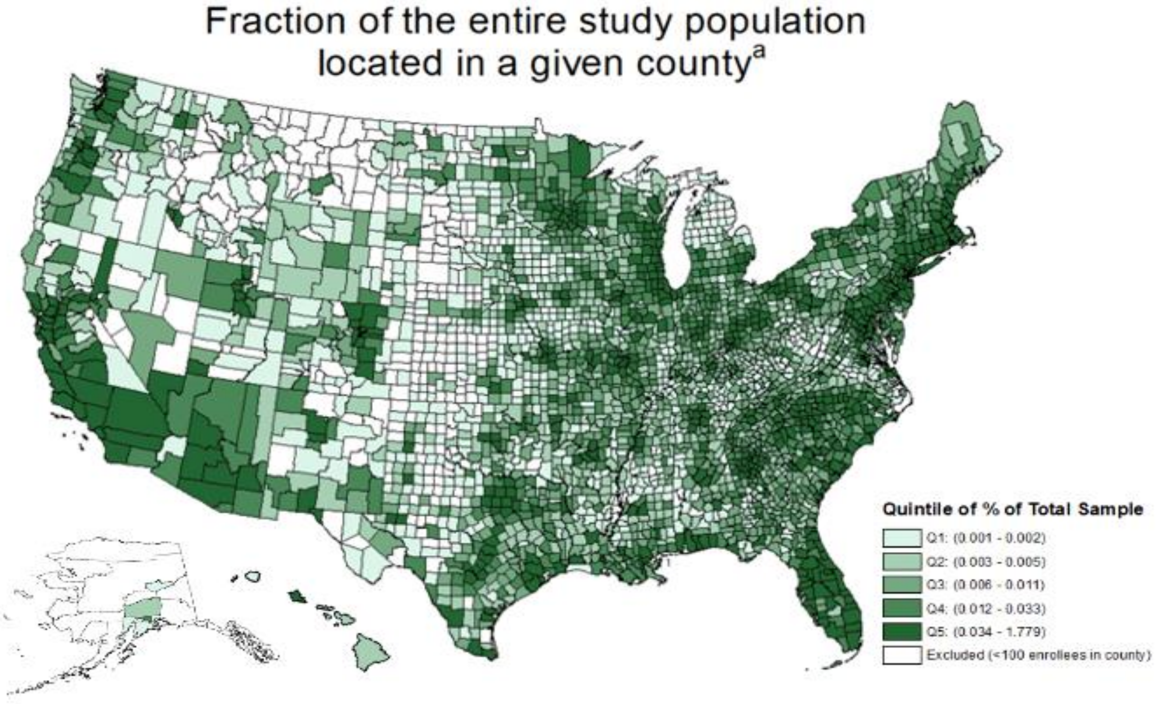

© 2021 Patel SY et al. *JAMA Network Open.*

[a] Numerator is the number of enrollees in each county. Denominator is the total number of enrollees in our study population.

Given the large increase in unemployment during the pandemic and differential loss of insurance and drop out of our data in 2020, we compared plan disenrollment in 2020 vs. prior years and find it is similar.

	Coverage in January & February (2 months)	Number of Enrollees with Coverage in January & February who Remain in Final Cohort	% Remaining in Final Cohort
2018	18,165,611	16,758,677	92.3%
2019	18,078,899	16,675,196	92.2%
2020	17,780,802	16,368,851	92.1%

Measuring county-level telemedicine use

During the pandemic, Medicare expanded the outpatient services that were eligible for telemedicine reimbursement and most private insurers replicated these changes.[2] We defined outpatient visits using Common Procedural Terminology (CPT) codes, excluding all CPT codes that were specific for clinical settings outside of clinician offices (e.g., emergency departments, hospital inpatient, nursing home or dialysis facility codes). CPT codes that are specific to clinical settings outside of clinician offices that were excluded from our analysis included: G0459, G0425-7, G0508-9, G0406-8, 99356-7, 99480, 99475-9, 99471-2, 99468-9, 99347-50, 99341-5, 99334-7, 99327-8, 99324-6, G9685, 99315-6, 99310, 99304-9, 99291-2, 99281-5, 99238-9, 99235-6, 99217-20, 94002-4.

We defined telemedicine visits (audio-video or audio only) as visits with modifier codes GT, GQ, or 95 or telemedicine specific CPT codes 99441-99443.[3,4] The remaining visits were categorized as in-person visits. Unfortunately, we cannot accurately distinguish between audio-only or video telemedicine visits. Given the low numbers of audio-only telemedicine visit CPT codes observed, we believe these codes were underutilized during the pandemic. The table below provides the number of outpatient visits by type during the 17-week pandemic period (March 18-July 14, 2020).

In-person Visits	Telemedicine Visits (audio-only & video)	Audio-Only Visits	Total Visits
17,546,289 (73%)	6,379,552 (27%)	681,657 (3%)	23,925,841

We assessed county-level telemedicine use during the 17-week pandemic period (March 18-July 14, 2020) using the percent of total visits delivered via telemedicine (defined as the number of telemedicine

© 2021 Patel SY et al. *JAMA Network Open.*

visits divided by the number of total visits). We also considered per capita telemedicine use as an outcome measure. However, we focused on percentage of total visits delivered via telemedicine due to its interpretability, because both outcome measures were highly correlated (p=0.81), and findings were similar across the two outcomes.

County-level Characteristics

We measured county-level characteristics using the most recently available data from the US Census (2019) Area Health Resource Files (2018). Due to strong correlation between medical doctors and advanced practice registered nurses (p=0.77), we combined them into a single measure of providers. Other health care system measures were considered, including the number of nurse practitioners and community mental health centers per capita, but were eliminated due to very high correlation with included measures.

We divided all measures into tertiles for meaningful interpretation. Given the numerous covariates and high-dimensional nature of variables, we assessed for positivity violations. Positivity violations occur when certain subgroups in a sample rarely or never receive some treatments of interest. The resulting sparsity in the data may increase bias with or without an increase in variance and can threaten valid inference.[6] We assessed positivity violations by comparing the propensity score distribution for each predictor variable in its three-category (tertile) form. The propensity score was measured via a series of logistic regressions (one for each predictor variable) with the three-category predictor as the outcome and the remaining covariates as predictors. We addressed positivity violations by treating these variables as binary indicator variables.

Statistical Analysis

To measure the association between these county characteristics and telemedicine use, we used the machine learning procedure, targeted maximum likelihood estimation. In contrast with standard ordinary least squares regression, targeted maximum likelihood estimation has two main features we highlight here. Firstly, it does not rely on the correct specification of a parametric regression and instead flexibly leverages machine learning in a nonparametric model. Secondly, targeted maximum likelihood estimation incorporates information from the propensity score function (i.e., the probability of a community factor

given other covariates).[6,7] All estimators were constructed in the R programming language, using the SuperLearner[8] and tmle[9] packages. As part of our targeted maximum likelihood procedure, we used ensembles (i.e., collections of algorithms) to estimate the outcome regression and propensity score regression. The ensembles included the following methods: random forests (minimum node sizes of 50, 100, 150, and 200 counties), artificial neural networks (weight decay parameter 0.01), generalized additive models, generalized linear models, NNLS, and extreme gradient boosting.

We estimated two sets of analyses, one using cumulative COVID-19 cases during the first 30 days of the pandemic and another during the first 17 weeks. Only those county characteristics in Table 1 were included in the analysis. As a sensitivity analysis, we compared targeted maximum likelihood estimates to ordinary least squares regression estimates, and results were comparable in both magnitude and direction. Given the community factors associated with telemedicine uptake could vary among commercially insured and Medicare Advantage enrollees, in a second sensitivity analysis, we repeated the analyses for each population and found results were comparable to the main findings in both magnitude and direction.

© 2021 Patel SY et al. *JAMA Network Open.*

eReferences

1. Centers for Medicare & Medicaid Services. President Trump expands telehealth benefits for Medicare beneficiaries during COVID-19 outbreak. Newsroom. Published March 17, 2020. https://www.cms.gov/newsroom/press-releases/president-trump-expands-telehealth-benefits-medicare-beneficiaries-during-covid-19-outbreak.

2. Centers for Medicare & Medicaid Services. List of telehealth services. Updated April 30, 2020. Accessed July 15, 2020. https://www.cms.gov/Medicare/Medicare-General-Information/Telehealth/Telehealth-Codes.

3. UnitedHealthcare. Coverage summary: telemedicine/telehealth services. Published 2017. Accessed Aug 29, 2017. https://www.unitedhealthcareonline.com/ccmcontent/ProviderII/UHC/en-US/Assets/ProviderStaticFiles/ProviderStaticFilesPdf/Tools%20and%20Resources/Policies%20and%20Protocols/UnitedHealthcare%20Medicare%20Coverage/Telehealth_and_Telemedicine_UHCMA_CS.pdf.

4. Center for Medicare and Medicaid Services. Telehealth services. Published 2016. Accessed Aug 29, 2017. https://www.cms.gov/Outreach-and-Education/Medicare-Learning-Network-MLN/MLNProducts/downloads/TelehealthSrvcsfctsht.pdf.

5. Petersen ML, Porter KE, Gruber S, Wang Y, van der Laan MJ. Diagnosing and responding to violations in the positivity assumption. *Stat Methods Med Res*. 2012;21(1):31-54. doi:10.1177/0962280210386207

6. Schuler MS, Rose S. Targeted maximum likelihood estimation for causal inference in observational studies. *Am J Epidemiol*. 2017;185(1):65-73. doi:10.1093/aje/kww165

7. van der Laan MJ, Rose S. *Targeted Learning: Causal Inference for Observational and Experimental Data*. New York, NY: Springer-Verlag New York; 2011.

8. Polley EC, van der Laan MJ. SuperLearner: Super Learner Prediction. (Package version 2.0-10). http://cran.rproject.org/web/packages/SuperLearner/index.html. Published 2013. Accessed November 15, 2020.

9. Gruber S, van der Laan MJ. TMLE: an R package for targeted maximum likelihood estimation. *J Stat Softw*. 2012;51(13):1–35.

© 2021 Patel SY et al. *JAMA Network Open.*
